# The Gut Barrier, Intestinal Microbiota, and Liver Disease: Molecular Mechanisms and Strategies to Manage

**DOI:** 10.3390/ijms21218351

**Published:** 2020-11-07

**Authors:** Julio Plaza-Díaz, Patricio Solís-Urra, Fernando Rodríguez-Rodríguez, Jorge Olivares-Arancibia, Miguel Navarro-Oliveros, Francisco Abadía-Molina, Ana I. Álvarez-Mercado

**Affiliations:** 1 Children’s Hospital of Eastern Ontario Research Institute, Ottawa, ON K1H 8L1, Canada; jrplaza@ugr.es; 2 Department of Biochemistry and Molecular Biology II, School of Pharmacy, University of Granada, 18071 Granada, Spain; 3 Instituto de Investigación Biosanitaria IBS.GRANADA, Complejo Hospitalario Universitario de Granada, 18071 Granada, Spain; 4 Faculty of Education and Social Sciences, Universidad Andres Bello, Viña del Mar 2531015, Chile; patricio.solis.u@gmail.com; 5 IRyS Research Group, School of Physical Education, Pontificia Universidad Católica de Valparaíso, Valparaíso 2374631, Chile; fernando.rodriguez@pucv.cl (F.R.-R.); jorge.olivares.ar@gmail.com (J.O.-A.); 6 Escuela de Pedagogía en Educación Física, Facultad de Educación, Universidad de las Américas, Santiago 8370035, Chile; 7 BioCritic. Group for Biomedical Research in Critical Care Medicine, 47005 Valladolid, Spain; miguelno@ugr.es; 8 Institute of Nutrition and Food Technology “José Mataix”, Center of Biomedical Research, University of Granada, Avda. del Conocimiento s/n. 18016 Armilla, Granada, Spain; fmolina@ugr.es; 9 Department of Cell Biology, School of Sciences, University of Granada, 18071 Granada, Spain

**Keywords:** liver disease, intestinal barrier, intestinal permeability, microbiota

## Abstract

Liver disease encompasses pathologies as non-alcoholic fatty liver disease, non-alcoholic steatohepatitis, alcohol liver disease, hepatocellular carcinoma, viral hepatitis, and autoimmune hepatitis. Nowadays, underlying mechanisms associating gut permeability and liver disease development are not well understood, although evidence points to the involvement of intestinal microbiota and their metabolites. Animal studies have shown alterations in Toll-like receptor signaling related to the leaky gut syndrome by the action of bacterial lipopolysaccharide. In humans, modifications of the intestinal microbiota in intestinal permeability have also been related to liver disease. Some of these changes were observed in bacterial species belonging *Roseburia*, *Streptococcus*, and *Rothia*. Currently, numerous strategies to treat liver disease are being assessed. This review summarizes and discusses studies addressed to determine mechanisms associated with the microbiota able to alter the intestinal barrier complementing the progress and advancement of liver disease, as well as the main strategies under development to manage these pathologies. We highlight those approaches that have shown improvement in intestinal microbiota and barrier function, namely lifestyle changes (diet and physical activity) and probiotics intervention. Nevertheless, knowledge about how such modifications are beneficial is still limited and specific mechanisms involved are not clear. Thus, further in-vitro, animal, and human studies are needed.

## 1. Introduction

Gut and liver are communicated by a bidirectional connection across the portal vein, the biliary tract, and the systemic circulation. This relation encloses reciprocal cellular and molecular interactions in which diet, genetic, and environmental cues are key players [1]. The bile acids also act as essential mediators. The portal vein allows the nutrients and bacterial compounds carriage and their metabolites through the intestinal lumen across the gut barrier to the liver, which contributes to homeostasis under healthy physiological conditions [2]. In this regard, the gut–liver axis is becoming an important variable in the pathogenesis of liver disease referring to the bidirectional relationship between the gut and its microbiota and the liver, resulting from the integration of signals generated by dietary, genetic, and environmental cues [3,4]. The integrity of the gut epithelium, immune defense in the gut and liver, and the composition of the microbiota all appear to play an integrated role in maintenance of health and balance in the gut–liver axis [5].

Indeed, loss of balance in microbial population and function, or dysbiosis, provokes the disruption of the intestinal barrier tight-junctions (TJs); this morphological alteration leads to increased intestinal permeability (also known as “leaky gut”) and an increment in the portal influx of bacteria or their products to the liver [4]. It is known that an increment in permeability in the intestine and the translocation of bacteria could facilitate that microbial metabolites getting to the liver, leading to the impairment of bile acids metabolism and the promotion of both systemic inflammation and gut dysmotility [6]. Indeed, non-alcoholic fatty liver disease (NAFLD) pathophysiology has been connected to minor microbial diversity and deteriorated intestinal barrier, revealing the host to constituents of bacteria and promising pathways related to inflammation via Toll-like receptors (TLRs) signaling and immune defense [7]. Moreover, this activation of inflammation in hepatocytes encourages development from simple steatosis to non-alcoholic steatohepatitis (NASH). This advance also may be described through the cytotoxicity related to the rise in main fecal bile acids, the primary/secondary fecal bile acids ratio, and plasma and hepatic bile acids concentrations [8]. In addition, patients with NASH present augmented intestinal permeability and raised plasma lipopolysaccharide (LPS), which may also contribute to liver sinusoidal endothelial cells pro-inflammatory action [9,10,11].

Nowadays, the gradual assumption of the Western lifestyle, including transformations in nutritional habits, physical inactivity, and alcohol abuse, has led to an increment in the frequency and incidence of NAFLD and alcoholic liver disease (ALD) as well as related metabolic disorders [12,13,14]. Both ALD and NAFLD include a wide range of hepatic lesions from asymptomatic steatosis to strict complications, e.g., steatohepatitis, fibrosis, cirrhosis, and hepatocellular carcinoma [15], being the final-stage liver disease, one of the most common causes of morbidity and mortality worldwide [16].

To date, the underlying mechanisms associated with gut permeability and liver disease development are not deeply deciphered, although knowledge points to the participation of intestinal microbiota in the pathogenesis of these diseases. Patients with NAFLD show fewer magnitudes of Bacteroidetes and greater amounts of *Prevotella* and *Porphyromas* than healthy subjects as well as elevated concentrations of *Lactobacillus*, *Escherichia,* and *Streptococcus* and diminished levels of *Ruminococcaceae* and *Faecalibacterium prausnitzii* [16]. Other authors have also described an increase in the Firmicutes/Bacteroidetes ratio [17,18]. Such increment might lead to elevated activity of TLRs and nucleotide-binding oligomerization domain (NOD) pathways, which could result in an alteration of the tight junction multiprotein complexes leading to increased gut permeability [19].

Bacterial translocation, defined as “translocation of bacteria and/or bacterial products (LPS, peptidoglycans, muramyl-dipeptides, bacterial DNA, etc.) from the gut to mesenteric lymph nodes” [20,21], is a physiological process in healthy conditions and crucial for host immunity [20]. Pathological bacterial translocation has been linked with an important role in the pathogenesis of liver diseases and complications, especially in cirrhosis [20], and could be influenced by bacterial overgrowth, intestinal barrier and gut-associated lymphatic tissue deficiencies, and an inappropriate immune response to the presence of bacteria and/or bacterial products [20].

Concerning ALD, the alcohol intestinal metabolism makes a great quantity of toxic acetaldehyde that varies gut permeability and microbiota stability causing direct hepatocyte impairment. In long-time alcohol consumers, a modification of gut microbiota composition has been observed, particularly a rise of Gram-negative bacteria, which cause endotoxemia and hyper-activation in the immune system [22].

Consequently, intestinal microbiota and its bacterial metabolites promote the pathophysiology of liver diseases, being worse due to increased intestinal permeability.

The present review aims to highlight the mechanisms related to molecular pathways that promote and contribute to both liver disease development and liver disease progression and their relation with alterations in the intestinal barrier. We also summarize and discuss recent literature about potential therapeutic targets addressed to regulate the thickness of the mucosal layer, cell–cell junctions, and intracellular permeability to hinder the occurrence of these diseases. The following topics are reviewed: (a) intestinal barrier function and microbiota; (b) leaky-gut, gut microbiota relationship and liver disease; (c) current main strategies to treat liver disease; (d) drug therapies; (e) diet, liver disease, and gut permeability; (f) probiotics administration as a strategy for liver disease treatment; (g) physical exercise and liver diseases; and (h) further directions.

## 2. Intestinal Barrier Function and Microbiota

The intestinal epithelium is the main protection barrier used to preserve intestinal reliability and to safeguard the host from the ecosystem [23,24]. This epithelium comprises resistant, occlusive intracellular junctions called TJs [25]. TJs coexist at the apical side of cells and are constituted of signaling molecules, transmembrane proteins, and membrane-associated scaffolding proteins that anchor TJs to the actin cytoskeleton [26,27]. TJs transmembrane proteins include TJs-associated MARVEL proteins (TAMPs), claudins, and junctional adhesion molecules (JAMs) [24]. TAMPs, claudins, and JAMs attach scaffolding proteins, such as zonula occludens 1 (ZO-1), ZO-2, and ZO-3, which connect them to the actin cytoskeleton [26,27]. Additionally, the intestinal epithelium is reinforced through a dense film of mucus that comprehends very glycosylated glycoproteins titled mucins (MUCs), principally created by specific epithelial cells recognized as goblet cells [28]. MUC2 is predominantly secreted, whereas MUC1, MUC3, and MUC4 are transmembrane proteins that act in a double system in the colon including an interior, thick layer with limited microbes and an external layer where the colonic microbiota resides [29]. In addition, the mucosal epithelium is in stable interaction with luminal contents and the enteric microbiota, which is variable and dynamic [23]. Intestinal microbiota refers to the biological community of commensal, symbiotic, and pathogenic microorganisms that cohabit on and within a host [30]. This could include bacteria, archaea, fungi, protozoa, and, more recently, viruses [31]. Today, bacteria remain in the attention due to the absence of well-organized approaches to report the other aforementioned organisms [32] being the major taxa present in intestinal microbiota Firmicutes and Bacteroidetes, whose amounts appear to persist remarkably continual during the life [33,34]. The microbiome comprises all of the genetic material within a microbiota. This is also referred to as the metagenome of the microbiota [35,36,37].

Currently, the microbiota is involved in the networks among the microbial populations that exist in our gut and certain conditions, physiological as well as pathological [35]. Among others, some extensively reported examples of pathological conditions in which microbiota play a role are cardiovascular disease [32], obesity-associated comorbidities, type 2 diabetes (T2D), and NAFLD [38,39].

In addition, short-chain fatty acids (SCFAs) are a significant basis of energy for enterocytes and are crucial signaling compounds for the preservation of gut health. In addition, SCFAs can arrive at the systemic circulation and interrelate with cell receptors in peripheral tissues [39]. Acetate is an important product of carbohydrate fermentation of most anaerobic bacteria, whereas propionic and butyric acids are produced from carbohydrate or protein fermentation through a different subdivision of bacteria [40,41]. Finally, intestinal permeability could permit some microbial components, bacteria, and metabolites to translocate into the liver [42].

## 3. Leaky Gut, Gut Microbiota Relationship and Liver Disease

Liver disease causes approximately 2 million deaths per year worldwide, 1 million due to difficulties of cirrhosis and 1 million due to viral hepatitis and hepatocellular carcinoma (HCC) [43]. Among others, liver diseases comprise several related diseases such as NAFLD, NASH, cirrhosis, HCC, viral hepatitis, and autoimmune hepatitis (AIH). Especially in childhood, NAFLD is experiencing a worldwide increment due to lifestyle changes such as junk food consumption, becoming a global public health problem. Indeed, one-fifth of the world’s children are already affected [44].

NAFLD is characterized by hepatic fat accumulation and can evolve to more severe and irreversible illnesses such as NASH, fibrosis, cirrhosis, or HCC. One of the current treatments for NAFLD is a restricted diet and weight loss through physical activity [45,46]. Exercise can reduce significantly the hepatic fat in NAFLD [47]. Likewise, 12 months of a regime alteration would reduce the fat and enzymatic activity in the liver in adults with NAFLD [48]. Several treatments based on herbal medicines also show improvements, although experiments have not been realized in humans yet. Some examples include piceatannol [49], a stilbenoid metabolite of resveratrol with antioxidant and anti-inflammatory effects on HepG2 hepatocytes, and MIT [50], an herbal formula comprising *Ephedra sinica*, *Panax ginseng*, and *Alisma orientale*, which has ameliorated NAFDL in mice.

NASH is a more strict type of liver disease than NAFLD, in which, besides steatosis, liver inflammation is observed [51,52]. Hernandez et al. shed light about the possibility that hypoxia could contribute to its progression in a patient with obstructive sleep apnea syndrome [53].

A key point to have in mind is the need for metabolic markers to elucidate which patients with early steatosis will suffer NAFLD and which will progress to an advanced form of liver disease; in this regard, phosphoglucose isomerase, a protein secreted in serum/plasma described in preclinical and clinical studies, is a promising fatty liver disease marker to discern between NAFLD and NASH [54,55]. Alcohol ingestion is one of the main causes of liver cirrhosis [56]. This causes the evolution of many symptoms such as steatosis, inflammation, and fibrosis and nowadays is the 11th most common reason for mortality globally, while HCC is the 16th prominent origin of death [56]. In fact, HCC is one of the most common malignant tumors and has an elevated yearly frequency and mortality [57]. Combined both diseases cause 3.5% of total deaths worldwide [43].

AIH is a necroinflammatory disease whose explanations remain unclear and attacks both children and adults of all ages [58,59]. Its incidence is estimated at 1–2 per 100,000 persons/year, while prevalence ranges 10–30 per 100,000 persons [60,61].

Pathological conditions such as toxins or gut inflammation can negatively affect barrier permeability, which may favor the translocation of luminal bacteria and their products (termed pathogen-associated molecular patterns (PAMPs)) [62]. The recognition of PAMPs by TLRs is a key point of the innate immune system and enables it to rapidly respond against invading pathogens [63]. In addition, most of the venous blood from the intestinal tract is drained into the portal circulation. The liver is therefore the first organ in the body to have contact with PAMPs [62]. Chronic exposure to increased levels of PAMPs has been linked to disease progression during early stages and infectious complications during late stages of liver disease [62]. PAMPs release also affects other organs, including the brain and kidney [64,65]. In this line, fermentation of protein and amino acids by gut bacteria can generate excessive amounts of potentially toxic compounds such as ammonia [66]. In the setting of the intestinal barrier and immune dysfunction, these byproducts are involved in the pathogenesis of complications of liver cirrhosis including hepatic encephalopathy [67]. Ammonia-rich blood reaches the liver via the portal circulation to be detoxified [68], but, when hyperammonemia occurs, the expression of microbial pattern recognition receptors such as TLRs is also upregulated, activating the immune response [69].

The liver controls systemic metabolism and the dissemination of compounds by the human intestine and, additionally, controls several hormones and immune responses. The interaction among the liver and the intestine is assisted by bile acids, which mediate in the incorporation of vitamins and dietary fats and act as ligand-binding for receptors such as farsenoid X receptor (FXR) and G-protein-coupled bile acid receptor 1 (or TGR5), which control the enterohepatic distribution [70]. On the other hand, chronic ethanol consumption causes endotoxemia, the increase of LPS, and hepatic inflammation as well as the development of the Gram-negative bacteria, especially Proteobacteria [71]. Hence, microbe-derived compounds and the signaling routes they influence could be implied in the progress of liver disease, especially NAFLD [72]. Below, we detail reported studies concerning leaky-gut, gut microbiota, and the mechanisms involved in developing liver diseases.

### 3.1. Animal Studies

In a study performed by Llopis and colleagues (2016), the effect of the transplantation of human gut microbiota from alcoholic donors with or without alcoholic hepatitis to germ-free and conventional mice was evaluated. Transplanted mice holding the intestinal microbiota from severe alcoholic hepatitis donors generated more inflammation in the liver with an augmented amount of liver T lymphocytes, enhanced gut permeability, hepatic necrosis, and bacterial translocation compared with those mice transplanted with gut microbiota from alcoholic donors without hepatitis. Key deleterious species were associated with recipients of alcoholic hepatitis microbiota. Conversely, *Faecalibacterium* (associated with anti-inflammatory properties) was observed in the mice group that received microbiota from donors without alcoholic hepatitis [73].

On the other hand, distinctive models of liver disease (i.e., obesity, cholestasis, toxic, and alcohol) demonstrated limited relationships in their gut microbiota [74]. For instance, an injury in the cholestatic liver stimulated by ligation of the usual bile duct and toxic liver injury produced through the carbon tetrachloride injection in mice produced an increment on intestinal permeability and bacterial translocation. Besides, carbon tetrachloride administration resulted in a rise in the Firmicutes and Actinobacteria relative abundance compared with control mice [74].

Changes in microbiota and their relationship with fibrosis were evaluated in mice treated with standard or HFD diet and subjected to carbon tetrachloride or bile duct ligation treatment. The results from this study reveal a higher fibrosis degree and bacterial translocation rate; increased Gram-negative bacteria, especially Proteobacteria in mice fed with HFD; and bile duct ligation. Given these findings, the authors concluded that dietary habits and intestinal Gram-negative bacteria, especially that produce endotoxin, could precipitate liver fibrogenesis [75]. It has been also reported that products in microbial translocation trigger an hepatic inflammatory response that contributes to steatohepatitis [76]. Specifically for ALD, TNF-receptor I (TNFRI) mutant mice were safeguarded from gut barrier dysfunction [77]. In addition, recovery of this receptor on gut epithelial cells caused augmented gut permeability and liver disease, suggesting that enteric TNFRI stimulates gut barrier dysfunction and is a critical intermediary of this disease [76]. Seo et al. demonstrated that the administration of *Roseburia* in ALD murine models ameliorated hepatic steatosis and inflammation, improving the gut ecosystem and preventing leaky gut. The suggested mechanisms were that recognition of TLR5, regained intestinal barrier integrity because of the upregulation of the TJ protein occluding, increased IL-22 expression, and the restoration of islet-derived protein 3-gamma [78].

The innate immunity assessment and Western-lifestyle diet in NAFLD progression were evaluated in Nlrp3^−/−^ and wild-type mice fed with a Western-lifestyle diet and drinking water with fructose or a chow diet. Knock-out animals for Nlrp3 treated with Western-lifestyle habits showed dysregulation of the response in intestinal immune with reduced expression of antimicrobial peptides, augmented gut permeability, and the incidence of a dysbiotic microbiota, which led to translocation of bacteria and augmented TLR4 and TLR9 hepatic expression. After antibiotic treatment, these changes were abridged, and unfavorable effects were reestablished [79].

Liver cirrhosis is related to bacterial translocation and endotoxemia. Two rodent models of increased bacterial translocation were used: CCl_4_-induced ascitic cirrhosis and two-day portal vein–ligated animals. Bacterial translocation was detected in 40% of the animals with cirrhosis. Both animal models have shown decreased intestinal Paneth cell α-cryptdin 5 and 7 expression [80]. Cirrhotic mice have shown that *Staphylococcaceae*, *Lactobacillaceae*, and *Streptococcaceae* were related with brain and systemic inflammation, and ammonia [81].

Importantly, hepatocytes can be an uninterrupted focus of microbial products [82]. Interestingly, PAMPs can trigger Kupffer cells to start hepatocyte impairment following the administration of alcohol [83]. Fatty liver progress seems to necessitate intestinal microbiota, but other microbiota-associated aspects might be a prerequisite for the liver disease pathogenesis in animal models, especially for NAFLD [72]. LPS reduction and intestinal TJs restoration might be an innovative therapeutic approach for the treatment of liver fibrosis expansion in NASH [84]. A study by Tedesco and colleagues (2018) used mice with Mdr2 gene disruption to regulate how faults in the liver and microbiota alteration provide the production of IL17 through intrahepatic γδ T cells. Mdr2^–/–^ mice had collagen accumulation in the hepatic bile ducts, fibrosis, and increased serum levels of IL17 compared with control mice. Fecal samples from Mdr2^–/–^ mice were enriched in *Lactobacillus*, liver tissues were enriched with *Lactobacillus gasseri* as well as an increment of intestinal permeability compared with control mice was observed. [85]. In addition, germ-free mice colonized with intestinal microbiota from two-week-old infants born to mothers with normal-weight or with obesity were compared to evaluate the effects of early colonization. Mice with stool microbes from obese mothers showed augmented gut permeability, abridged macrophage phagocytosis, and diminished production of cytokine indicative of impaired macrophage function, and histological signs of periportal inflammation, similar to pediatric cases of NAFLD. These results require functional data accompanying a causal part of maternal obesity-associated infant dysbiosis in NAFLD and childhood obesity [86].

### 3.2. Human Studies

Several studies have shown that patients with ALD or cirrhosis present augmented gut permeability, which might be a significant component in liver disease progression [87,88,89]. However, serum endotoxin was augmented in only 42.1% (8/19) of the patients with NASH [90] and a meta-analysis showed that only 39.1% of patients with NAFLD (*n* = 128) had augmented gut permeability [91]. However, it is significant to note that these outcomes are based on a moderately small number of patients [72].

Another study reported changes in the portal and systemic circulation of patients with cirrhosis. Such changes could be produced by the peripheral vasodilatation due to an overproduction of nitric oxide (NO). In fact, levels of NO correlated with the severity of liver disease and the activation of its synthetic enzymes (iNOS, eNOS, and nNOS) is mostly due to the effects of LPS and the released cytokines [92].

Concerning pediatric NASH patients, intestinal microbiota alterations have been observed. With respect to children with obesity or NAFLD, greater quantities of *Prevotella* and Bacteroidetes were found matched to healthy controls [93]. In addition, Korean patients with ALD have shown a strong association between the abundance of the butyrate-producing genus *Roseburia* and decreased values in the Alcohol Use Disorders Identification Test (a method of screening for excessive drinking and alcohol use disorders) [78].

In addition, in ALD patients, the antibacterial potency of mucosal-associated invariant T cells was impaired because of a significant interaction with intestinal microbiota and microbial products, signifying that the “leaky” gut observed in ALD drives the dysfunction of cells and explains in part the susceptibility to infection in the ALD patients [94]. In addition, augmented gut permeability, derangement of the intestinal microbiota, and translocation of bacteria occurred in AIH patients, which is associated with the gravity of the disease [95].

Recent analyses have shown that gradual ALD at early disease stages is related to duodenal mucosal-associated dysbiosis and raised microbial translocation; remarkably, such alterations were not connected with augmented intestinal permeability. The bacterial species related to these changes include *Streptococcus*, *Shuttleworthia*, and *Rothia* [96].

On the other hand, fecal microbiota transplantation is the replacement of useful bacteria from the stool of healthy donors into the gastrointestinal tract of patients to restore the stability of the gut microbiota [32,97]. Patients with NAFLD were enlisted and randomly divided into either an allogenic or autologous fecal microbiota transplantation. Allogenic fecal microbiota transplantation patients with elevated small intestinal at reference had a significant decrease six weeks after transplantation [98]. Subjects with chronic liver disease and a disease-free control group undergoing routine endoscopy experienced a duodenal biopsy to evaluate duodenal mucosa-associated microbiota. *Streptococcus*-affiliated lineages were associated with liver disease patients and there were opposite associations between microbial diversity and both augmented small intestinal permeability and serum alanine aminotransferase in patients with chronic liver disease [99].

A recent study has shown that intestinal microbiota of AIH patients is reduced in its diversity and have changes in species belonging *Streptococcus*, *Veillonella*, *Klebsiella*, and *Lactobacillus* compared with healthy controls [100]. A pilot study with chronic liver disease patients was performed to analyze changes at different stages of liver disease [101]. The first study reported intestinal microbiota differences related to the grading of AIH activity but not to the stage of fibrosis; however, this pilot study indicated that changes in *Veillonella* and *Streptococcus* are aggravated with progressing chronic liver disease severity [101].

Cirrhotic patients have shown augmented levels of *Veillonella*, *Megasphaera*, *Dialister*, *Atopobium*, and *Prevotella*. Gene pathways related to sugar and amino acid metabolism were highly abundant in cirrhosis duodenal microbiota, and functional modules involved in bacterial motility proteins and secretion system were overrepresented in controls [102]. Fecal samples of patients with chronic hepatitis B and patients with hepatitis B virus-related cirrhosis were compared with fecal samples of healthy subjects to analyze the *Bifidobacterium* presence. *Bifidobacterium dentium* and *Bifidobacterium catenulatum/Bifidobacterium pseudocatenulatum* were detected less frequently in hepatitis B virus-related cirrhosis patients. Intestinal microbiota composition of patients with chronic hepatitis B and patients with hepatitis B virus-related cirrhosis was modified with a shift from beneficial species to opportunistic pathogens [103].

In a cross-sectional approach, the intestinal microbiota of patients with chronic hepatitis C virus infection and healthy controls was analyzed. This study showed that not only the stage of liver disease but also hepatitis C virus infection is related to decreased α-diversity and different microbial community patterns [104].

Results from the pediatric population with NAFLD have shown that, in early stages of the disease, plasma endotoxin concentrations are slightly raised, suggesting that gut barrier dysfunction could be present already in the initial phases of the disease [105]. Intestinal microbiota from children with NAFLD had lower α-diversity than those of control children, and higher quantities of *Prevotella copri* were related to more severe fibrosis [106]. Children with NASH have augmented serum LPS concentrations compared to controls, supporting the rationale that bacterial translocation products trigger the immune system [107].

Alcoholic hepatitis is a severe alcohol-associated liver disease with minimal treatment options [108]. A recent study by Duan et al. uncovered that *Enterococcus faecalis* is an important supporter in alcoholic hepatitis. This bacterium could be eradicated with a bacteriophage, suggesting new therapeutic approaches [109]. The diminution in the of *Roseburia* relative abundance is related to alcohol consumption in human cohorts. In contrast, the administration of *Roseburia intestinalis* ameliorates the experimental ALD in mice [78].

Finally, subjects with alcohol dependency established gut leakiness, which was related to greater depression scores, anxiety, and alcohol craving after three weeks of abstinence. Furthermore, subjects with augmented intestinal permeability also had different gut microbiota composition and activity [110]. In this line, a current study evaluated more than bacteria, including the mycobiota in patients with alcoholic hepatitis. The authors observed that *Candida* was the richest taxon in the fecal mycobiota of the alcohol group, while *Penicillium* controlled the mycobiome of nonalcoholic controls, and patients with alcoholic hepatitis had significantly augmented serum levels of anti-*Saccharomyces cerevisiae* antibodies matched to nonalcoholic controls and patients with alcohol use disorder [111].

## 4. Current Main Strategies to Treat Liver Disease

### 4.1. Drug Therapies

Many current drug therapies for liver disease are based on FXR agonists such as obeticholic acid. In humans, this drug prevents the progression from NASH to more severe complications [112]. In addition, peroxisome proliferator-activated receptors [113] and pregnane X receptor [114] have shown beneficial effects in NASH. Other potential drug treatments are based in stearyl-CoA desaturase-1, which transforms saturated fatty acids to monounsaturated fatty acid, or aramchol which has shown a fibrosis-stage reduction in a placebo-controlled, randomized trial during 52 weeks [115] (NCT02279524).

In liver cancer, Yes-associated protein 1 deletion in human umbilical vein endothelial cells by small interfering RNAs and verteporfin inhibited proliferation, migration, and angiogenesis becoming a promising target to slow down the illness [116].

Nowadays, the most effective treatment for AIH is corticosteroids [60,61]. In this line, Kirk et al. verified that prednisolone reduced mortality in the early active phase of the disease [117]. After them, Soloway’s and Summerskill’s groups established that the use of prednisolone and azathioprine blocks purine metabolism and DNA synthesis, evidencing that both drugs are equally effective in combination as the prednisolone monotherapy [118,119]. To note, none of the above-mentioned studies aimed to evaluate how the potential drugs tested affect gut permeability and/or microbiota integrity. Consequently, considering how alterations in the intestinal barrier as well as an altered microbiota profile have a strategic function in the progress of the liver disease, it is mandatory to assess these parameters in the future.

### 4.2. Diet, Liver Disease and Gut Permeability

Overnutrition is critical for the pathophysiology and development of liver diseases [120]. For instance, high-fat diet (HFD) can modulate the composition of the intestinal microbiota through the reduction of the protective intestinal bacteria and favoring the prevalence of opportunistic pathogenic products of Gram-negative bacteria, such as LPS [121]. The presence of LPS in portal circulation enables the binding to TLR4 and other co-receptors in the liver associated with inflammation, leading to NASH [122]. Regarding pathogenic HCC pathways, observational studies in humans and animals have linked specific dietary compounds and dietetic habits with the chance of liver cancer development by several mechanisms including dysbiosis [123]. Nuclear-FXR knockout mice progress macroscopically visible liver tumors after 15 months of Western diet feeding. Both Western diet intake and FXR deficiency caused hepatitis, gut dysbiosis, and reduced butyrate production, pointing to a role of dysbiosis-associated dysregulated bile acid synthesis in hepatic inflammation promotion, which, in turn, contributes to carcinogenesis [124]. Conversely, dietary soluble fibers are considered to have positive effects on health status since they are fermented by intestinal bacteria into SCFAs [125]. Alcohol administration caused gut leakiness in rats, which was associated with both endotoxemia and liver injury, while oats prevented these changes [126]. In addition, in rodents, fructose consumption produced leaky gut, microbiota changes, and hepatic inflammation/fibrosis as well as augmented protein cytochrome P450-2E1 levels, a nitroxidative stress marker in the liver. Ingestion of fructose significantly raised the levels of plasma bacterial endotoxin, possibly a consequence after reduced levels of intestinal TJs proteins [127]. In humans, the high hepatic fat content was analyzed. Subjects who received this kind of diet were evaluated. Subjects with high hepatic fat content developed an unfavorable intestinal microbiota composition, categorized through the lower *Faecalibacterium prausnitzii* amount and relatively more *Enterobacteria* than the low hepatic fat content group [128].

Excessive food intake as well as certain dietary patterns are strongly related to alterations in the intestinal barrier [13]. The interplay between diet-related liver disease and gut permeability has been mainly approached in intervention and dietary supplementation studies [129,130,131]. However, from our knowledge, the effect of a specific diet in the gut barrier has not been widely considered and only a few studies have been reported in the last years. In this regard, and consistent with other results, Biolato et al. revealed improvements in visceral obesity, weight, and serum transaminase profile in patients with NAFLD undergoing the Mediterranean or low caloric diet for 16 weeks. However, no significant modifications in intestinal permeability were observed by these authors [132]. On the contrary, individuals with obesity and with or without liver steatosis undertaking a weight-reduction schedule and a comprehensive lifestyle modification during 52 weeks reduced the increased permeability to the normal range, which highlights the link of gut permeability with not only body weight and insulin resistance but also fatty liver disease in the obesity context [133].

HFD occasioned a significantly augmented hepatic steatosis and inflammation in a sirtuin3 (SIRT3) knockout mice. The absence of SIRT3 accelerates intestinal microbial dysbiosis in mice after HFD with augmented *Desulfovibrio* and *Oscillibacter* and decreased *Alloprevotella* bacterial levels [134].

Finally, the properties of an elevated fiber diet and sodium butyrate on the Treg/Th17 and intestinal barrier role in experimental AIH were evaluated. Both treatments significantly decreased serum aminotransferases, *Escherichia coli* protein in the liver, and liver injury matched with the control group. Furthermore, the Treg/Th17 ratio and TJ proteins were augmented in both treatments [135].

### 4.3. Probiotics Administration As a Strategy for Liver Disease Treatment

Probiotics are defined as microorganisms that offer well-being benefits to hosts when dispensed in sufficient quantities [39]. Currently, probiotics are used to manage dysbiosis, restore the microbe diversity, and reestablish disturbed gut microbiota. However, particular tools have not been clarified yet [136,137]. For instance, in the case of *Lactobacillus* strain, *Lactobacillus rhamnosus* GG administration was tested to preserve the barrier role in ALD animal model resulting in and significantly ameliorated alcoholic steatohepatitis. This progress was related to the reduction of intestinal and liver oxidative stress markers and inflammation and preservation of intestinal barrier function [138]. Besides, cell-free supernatant from *Lactobacillus reuteri* ZJ617 was able to prevent the interruption of the intestinal barrier by preventing the influence of LPS through TLR4 activation [139]. *Lactobacillus rhamnosus* GG administration was also able to protect the NAFLD progression by reducing hepatic expression of IL-1β, IL-8R, and TNF-α; increasing LPS accumulation; and increasing beneficial bacteria in the NAFLD model provoked through a high-fructose regime [140]. A randomized controlled trial performed in NASH patients consisting in the administration of *Lactobacillus reuteri* plus prebiotics produced the decrease of steatosis, weight, waist circumference, and body mass index. This improvement occurred despite the lack of effects on gut permeability. The authors also found no amelioration in serum levels of LPS at the end of the intervention [129]. In addition, in humans suffering from chronic liver disease, the treatment with six bacterial species (three species of *Bifidobacterium,* two species of *Lactobacillus*, and *Streptococcus thermophilus*) was tested in six groups. Small intestinal bacterial overgrowth disappeared, stool counts of lactobacilli were correlated negatively with intestinal permeability, and no liver improvement was observed [141].

De Simone formulation, a multistrain probiotic preparation [39], was tested in a genetic dyslipidemia model in rats to evaluate its effect in the progression of steatohepatitis. De Simone formulation prevented the progress of histologic characters of mesenteric adipose tissue inflammation, ameliorated steatohepatitis, and abridged the range of aortic plaques acting in the FXR, peroxisome proliferator-activated receptor-γ, and vitamin D receptor [142]. Another study performed in a liver fibrosis rat model provoked by carbon tetrachloride evaluated the effects of *Saccharomyces boulardii,* finding decreased collagen type I alpha 1, alpha-smooth muscle actin, and transforming growth factor-beta hepatic expression. Remarkably, the treatment with *S. boulardii* was related to changes in intestinal permeability and the composition of fecal microbiota [143].

A double-blind phase 2 trial was performed in patients with NAFLD to test the effects of fructo-oligosaccharides, 4 g twice per day, plus *Bifidobacterium animalis* subspecies *lactis* BB-12 or placebo for in intestinal microbiota during 10–14 months. *Bifidobacterium* and *Faecalibacterium* were higher in the fecal samples from patients who received the synbiotic, whereas *Oscillibacter* and *Alistipes* species were decreased. Differences observed in the intestinal microbiota were not related to liver fat or markers of fibrosis [144].

### 4.4. Physical Exercise and Liver Disease

Physical exercise is one of the most favorable non-pharmacological methodologies for the prevention and management of several diseases including neurological, metabolic, and cardiovascular diseases [145]. A sedentary lifestyle is a significant chance factor in the progress of NAFLD and NASH [146], being a very common feature in NAFLD patients [147].

The potential mechanism by which physical exercise provides benefits on NAFLD is through the modification of body composition, reducing hepatic steatosis or intrahepatic fat, while the benefits might be evident even in the absence of weight loss [146,148,149]. Interestingly, the level of physical fitness is an important predictor, independently of adiposity, of the degree of hepatic steatosis, [146,148]. In addition, baseline values of physical fitness predict efficiency for lifestyle intervention to decrease hepatic steatosis in patients with NAFLD [148,150]. The mechanism linked to the benefits of exercise in NAFLD share common mechanisms related to metabolic syndrome as lipid oxidation, glucose control, and insulin metabolism and include reduction of the adiposity profile, improvement of inflammation, and immune parameters [146,148,149].

#### Exercise on Gut Barrier Permeability and Microbiome

Although it is well recognized that one of the immediate (acute) effects of physical exercise is intestinal permeability, the exercise effect on the immune system, and microbiota diversity suggests that exercise also provides long-term benefits for the intestinal barrier [151]. Interestingly, it has been established that intestinal microbiota fermentation determines the efficacy of exercise intervention in insulin sensitivity and glucose metabolism [149]. In this line, physical exercise effect on intestinal microbiota has been highlighted as responsible for modifying intestinal villi morphology, the enterohepatic circulation of bile acids, and the attenuation of gut symbiosis. Thus, physical exercise intervention could induce changes in the bile acid pool and modify microbiota diversity [146].

Accordingly, some authors have proposed that the reduction of intestinal permeability after chronic physical exercise may occur due to a greater abundance of the commensal bacterial population, the increment of Bacteriodetes/Firmicutes ratio, and an overall microbial diversity [152]. Additionally, physical exercise may trigger benefits in the gut mucus layer by saving microbes from holding to the intestinal epithelium and serving as a substrate for mucosa-associated bacteria (i.e., *Akkermansia muciniphila*). Exercise has also demonstrated an increased abundance of SCFAs, which has been shown to increase colonic epithelial cell propagation, providing benefits to intestinal barrier integrity [151] and an increase in butyrate, which promotes growth energy spending, insulin sensitivity recovery, and decreased adiposity. Notably, butyrate and other SCFAs also participate in the regulation of food intake, stimulating the production of satiety hormones and, in consequence, are implicated in glucose metabolism regulation and the improvement of gut barrier function. Moreover, elevated levels of butyrate induce intestinal T-reg cells regulation of the anti-inflammatory cytokines liberation and promotion of the gut barrier integrity [152]. All the above-mentioned is in line with extensive evidence that indicates the induction of alterations in the gene expression of intraepithelial lymphocytes and the effects of systemic inflammation associated with physical exercise [151,153,154,155].

In summary, physical exercise provides several benefits in the prevention and treatment of NASH and NAFLD. Such favorable effects imply the metabolic profile improvement and the reestablishment of intestinal microbiota composition, intestinal barrier integrity, immune system, and inflammation profile. Consequently, exercise should be considered as an important tool when approaching potential treatment options.

## 5. Further Directions

Currently, neither the liver disease pathophysiology nor the associated-microbiota alterations have been completely characterized. However, the incidence and prevalence of these diseases and their more severe complications as well as related metabolic disorders are expanding worldwide [12,13], which represents a global burden to the public health systems [156].

**Figure 1:**
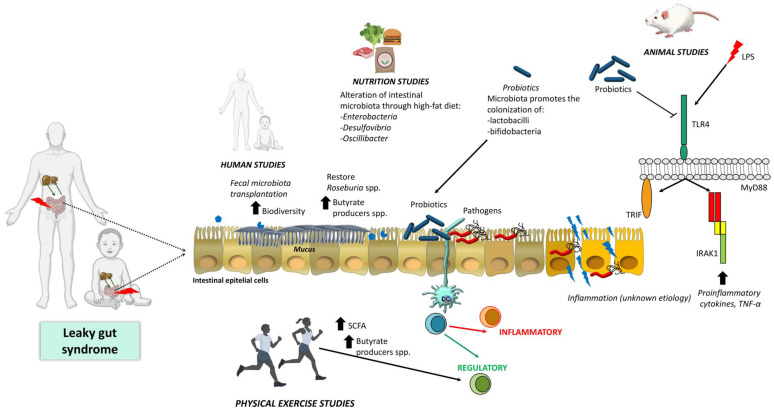
Main reported roles and mechanisms involving leaky gut and intestinal microbiota changes for the treatment of liver disease. Abbreviations: IRAK1, Interleukin 1 Receptor Associated Kinase 1; LPS, lipopolysaccharide; MyD88, Myeloid differentiation primary response 88; SCFA, short-chain fatty acids; TLR4, toll-like receptor 4; TNF-α, tumor necrosis factor-alpha; TRIF, TIR-domain-containing adapter-inducing interferon-β.

Progression from simple steatosis to NASH dramatically raises the possibilities of liver failure, cirrhosis, and HCC [157,158,159]. It is estimated that 10–15% of patients with NASH will develop HCC [157]. Weight loss via diet and routine modifications are positive commendations to ameliorate liver damage. Indeed, an ordinary weight loss of about 3% might decrease hepatic steatosis up to 10% or more [156]. In this line, physical exercise provides additional potential benefits to microbiota composition, gut barrier integrity, and metabolic profile, including reduction of adiposity profile and, improvement of inflammation and immune parameters [146,148,149], suggesting their suitability as a potential tool of treatment and prevention. In addition, long-term therapy with different drugs regulating transcription factors such as FXR [160,161], peroxisome proliferator-activated receptors [113], and pregnane X receptor [114] have shown beneficial effects in NASH. Nevertheless, a reduced number of strategies or recommendations to treat liver disease have to take into account the evidence that bacterial products such as metabolites and bacterial wall components into the circulation add to the pathogenesis of the liver disease. Figure 1 summarizes known mechanisms and the strategies developed to date involving leaky gut and intestinal microbiota changes to treat or ameliorate the progression of liver disease. Researchers usually miss the fact that the damage caused by microbiota products in the function of the intestinal barrier, reflected in increased permeability, is crucial for the development of these disorders. Thus, a deeper knowledge of the molecular mechanisms implied in the loosening of the TJs protein complexes favoring the leaky gut syndrome, as well as the bacterial or their metabolites profile implied, could increase the efficacy of existing therapies and shed light on important items currently unsolved such as: (a) which patients will progress toward the end-stage liver disease; (b) decipher the pathogenic mechanism(s) behind progression; and (c) provide patients at risk of disease progression with more effective pharmacological strategies.

It is worth noting that a broad field has opened since the dysregulated crosstalk between the peripheral organs and intestinal microbiota is implied not only in liver disease, but also in a wide number of pathologies.

For instance, dysfunctions in the bidirectional interaction between the brain and the gut (gut–brain axis) associated with alterations in the gut microbiome and intestinal permeability have been the subject of several recent extensive reviews and original studies [162,163,164,165,166,167,168,169]. These works report evidences of neurological conditions and disorders such as Alzheimer’s disease [163], autism spectrum disorder [164], Parkinson’s disease [165], stress-related psychiatric disorders [167], schizophrenia [168], major depressive disorder [166], and anorexia nervosa [169], related to intestinal permeability.

In conclusion, given the recent results pointing that both gut microbiota and intestinal permeability are deeply involved in several diseases, new knowledge leading to strategies able to ameliorate leaky gut (i.e., physical activity, diet, probiotics, etc.) are urgently needed. During the past decade, intestinal microbiome has emerged as an important liver disease modifier [170]; nevertheless, the knowledge about how such modifications occur is limited and it is not clear what specific mechanisms are involved in the reported benefits. Thus, more in-vitro, animal, and human studies are needed to get a comprehensive picture of the relationship between liver disease and intestinal microbiota.

## References

[1] Tripathi A., Debelius J., Brenner D.A., Karin M., Loomba R., Schnabl B., Knight R. (2018). The gut-liver axis and the intersection with the microbiome. Nat. Rev. Gastroenterol. Hepatol..

[2] Delzenne N.M., Knudsen C., Beaumont M., Rodriguez J., Neyrinck A.M., Bindels L.B. (2019). Contribution of the gut microbiota to the regulation of host metabolism and energy balance: A focus on the gut-liver axis. Proc. Nutr. Soc..

[3] Wiest R., Albillos A., Trauner M., Bajaj J.S., Jalan R. (2017). Targeting the gut-liver axis in liver disease. J. Hepatol..

[4] Albillos A., De Gottardi A., Rescigno M. (2020). The gut-liver axis in liver disease: Pathophysiological basis for therapy. J. Hepatol..

[5] Szabo G., Bala S., Petrasek J., Gattu A. (2010). Gut-liver axis and sensing microbes. Dig. Dis..

[6] Milosevic I., Vujovic A., Barac A., Djelic M., Korac M., Radovanovic Spurnic A., Gmizic I., Stevanovic O., Djordjevic V., Lekic N. (2019). Gut-Liver Axis, Gut Microbiota, and Its Modulation in the Management of Liver Diseases: A Review of the Literature. Int. J. Mol. Sci..

[7] Baffy G. (2019). Potential mechanisms linking gut microbiota and portal hypertension. Liver Int..

[8] Poeta M., Pierri L., Vajro P. (2017). Gut-Liver Axis Derangement in Non-Alcoholic Fatty Liver Disease. Children.

[9] Harte A.L., Da Silva N.F., Creely S.J., McGee K.C., Billyard T., Youssef-Elabd E.M., Tripathi G., Ashour E., Abdalla M.S., Sharada H.M. (2010). Elevated endotoxin levels in non-alcoholic fatty liver disease. J. Inflamm. Lond..

[10] Brun P., Castagliuolo I., Di Leo V., Buda A., Pinzani M., Palu G., Martines D. (2007). Increased intestinal permeability in obese mice: New evidence in the pathogenesis of nonalcoholic steatohepatitis. Am. J. Physiol. Gastrointest Liver Physiol..

[11] Wu J., Meng Z., Jiang M., Zhang E., Trippler M., Broering R., Bucchi A., Krux F., Dittmer U., Yang D. (2010). Toll-like receptor-induced innate immune responses in non-parenchymal liver cells are cell type-specific. Immunology.

[12] Cornide-Petronio M.E., Alvarez-Mercado A.I., Jimenez-Castro M.B., Peralta C. (2020). Current Knowledge about the Effect of Nutritional Status, Supplemented Nutrition Diet, and Gut Microbiota on Hepatic Ischemia-Reperfusion and Regeneration in Liver Surgery. Nutrients.

[13] Alvarez-Mercado A.I., Navarro-Oliveros M., Robles-Sanchez C., Plaza-Diaz J., Saez-Lara M.J., Munoz-Quezada S., Fontana L., Abadia-Molina F. (2019). Microbial Population Changes and Their Relationship with Human Health and Disease. Microorganisms.

[14] Trovato G.M., Catalano D., Martines G.F., Pirri C., Trovato F.M. (2013). Western dietary pattern and sedentary life: Independent effects of diet and physical exercise intensity on NAFLD. Am. J. Gastroenterol..

[15] Meroni M., Longo M., Dongiovanni P. (2019). Alcohol or Gut Microbiota: Who Is the Guilty?. Int. J. Mol. Sci..

[16] Adolph T.E., Grander C., Moschen A.R., Tilg H. (2018). Liver-Microbiome Axis in Health and Disease. Trends Immunol..

[17] Tsai M.C., Liu Y.Y., Lin C.C., Wang C.C., Wu Y.J., Yong C.C., Chen K.D., Chuah S.K., Yao C.C., Huang P.Y. (2020). Gut Microbiota Dysbiosis in Patients with Biopsy-Proven Nonalcoholic Fatty Liver Disease: A Cross-Sectional Study in Taiwan. Nutrients.

[18] Wang C., Zhu C., Shao L., Ye J., Shen Y., Ren Y. (2019). Role of Bile Acids in Dysbiosis and Treatment of Nonalcoholic Fatty Liver Disease. Mediat. Inflamm..

[19] Shama S., Liu W. (2020). Omega-3 Fatty Acids and Gut Microbiota: A Reciprocal Interaction in Nonalcoholic Fatty Liver Disease. Dig. Dis. Sci..

[20] Wiest R., Lawson M., Geuking M. (2014). Pathological bacterial translocation in liver cirrhosis. J. Hepatol..

[21] Berg R.D., Garlington A.W. (1979). Translocation of certain indigenous bacteria from the gastrointestinal tract to the mesenteric lymph nodes and other organs in a gnotobiotic mouse model. Infect. Immun..

[22] Malaguarnera G., Giordano M., Nunnari G., Bertino G., Malaguarnera M. (2014). Gut microbiota in alcoholic liver disease: Pathogenetic role and therapeutic perspectives. World J. Gastroenterol..

[23] Bermudez-Brito M., Plaza-Diaz J., Munoz-Quezada S., Gomez-Llorente C., Gil A. (2012). Probiotic mechanisms of action. Ann. Nutr. Metab..

[24] Chopyk D.M., Grakoui A. (2020). Contribution of the Intestinal Microbiome and Gut Barrier to Hepatic Disorders. Gastroenterology.

[25] Farquhar M.G., Palade G.E. (1963). Junctional complexes in various epithelia. J. Cell Biol..

[26] Van Itallie C.M., Anderson J.M. (2014). Architecture of tight junctions and principles of molecular composition. Semin. Cell. Dev. Biol..

[27] Luissint A.C., Parkos C.A., Nusrat A. (2016). Inflammation and the Intestinal Barrier: Leukocyte-Epithelial Cell Interactions, Cell Junction Remodeling, and Mucosal Repair. Gastroenterology.

[28] Cornick S., Tawiah A., Chadee K. (2015). Roles and regulation of the mucus barrier in the gut. Tissue Barriers.

[29] Johansson M.E., Ambort D., Pelaseyed T., Schutte A., Gustafsson J.K., Ermund A., Subramani D.B., Holmen-Larsson J.M., Thomsson K.A., Bergstrom J.H. (2011). Composition and functional role of the mucus layers in the intestine. Cell Mol. Life Sci..

[30] Peterson J., Garges S., Giovanni M., McInnes P., Wang L., Schloss J.A., Bonazzi V., McEwen J.E., Wetterstrand K.A., Deal C. (2009). The NIH human microbiome project. Genome. Res..

[31] Roy S., Trinchieri G. (2017). Microbiota: A key orchestrator of cancer therapy. Nat. Rev. Cancer.

[32] Sanchez-Rodriguez E., Egea-Zorrilla A., Plaza-Diaz J., Aragon-Vela J., Munoz-Quezada S., Tercedor-Sanchez L., Abadia-Molina F. (2020). The Gut Microbiota and Its Implication in the Development of Atherosclerosis and Related Cardiovascular Diseases. Nutrients.

[33] Huttenhower C., Gevers D., Knight R., Abubucker S., Badger J.H., Chinwalla A.T., Creasy H.H., Earl A.M., FitzGerald M.G., Fulton R.S. (2012). Structure, function and diversity of the healthy human microbiome. Nature.

[34] Faith J.J., Guruge J.L., Charbonneau M., Subramanian S., Seedorf H., Goodman A.L., Clemente J.C., Knight R., Heath A.C., Leibel R.L. (2013). The long-term stability of the human gut microbiota. Science.

[35] Plaza-Diaz J., Gomez-Fernandez A., Chueca N., Torre-Aguilar M.J., Gil A., Perez-Navero J.L., Flores-Rojas K., Martin-Borreguero P., Solis-Urra P., Ruiz-Ojeda F.J. (2019). Autism Spectrum Disorder (ASD) with and without Mental Regression is Associated with Changes in the Fecal Microbiota. Nutrients.

[36] Fernandez M.F., Reina-Perez I., Astorga J.M., Rodriguez-Carrillo A., Plaza-Diaz J., Fontana L. (2018). Breast Cancer and Its Relationship with the Microbiota. Int. J. Environ. Res. Public Health.

[37] Tenorio-Jimenez C., Martinez-Ramirez M.J., Del Castillo-Codes I., Arraiza-Irigoyen C., Tercero-Lozano M., Camacho J., Chueca N., Garcia F., Olza J., Plaza-Diaz J. (2019). Lactobacillus reuteri V3401 Reduces Inflammatory Biomarkers and Modifies the Gastrointestinal Microbiome in Adults with Metabolic Syndrome: The PROSIR Study. Nutrients.

[38] Mouzaki M., Comelli E.M., Arendt B.M., Bonengel J., Fung S.K., Fischer S.E., McGilvray I.D., Allard J.P. (2013). Intestinal microbiota in patients with nonalcoholic fatty liver disease. Hepatology.

[39] Plaza-Diaz J., Ruiz-Ojeda F.J., Gil-Campos M., Gil A. (2019). Mechanisms of action of probiotics. Adv. Nutr..

[40] Louis P., Flint H.J. (2017). Formation of propionate and butyrate by the human colonic microbiota. Environ. Microbiol..

[41] Granado-Serrano A.B., Martín-Garí M., Sánchez V., Solans M.R., Berdun R., Ludwig I.A., Rubio L., Vilaprinyo E., Portero-Otín M., Serrano J. (2019). Faecal bacterial and short-chain fatty acids signature in hypercholesterolemia. Sci. Rep..

[42] Starkel P., Schnabl B. (2016). Bidirectional Communication between Liver and Gut during Alcoholic Liver Disease. Semin. Liver Dis..

[43] Asrani S.K., Devarbhavi H., Eaton J., Kamath P.S. (2019). Burden of liver diseases in the world. J. Hepatol..

[44] World Health Organization (2018). Taking Action on Childhood Obesity.

[45] Nourian M., Askari G., Golshiri P., Miraghajani M., Shokri S., Arab A. (2020). Effect of lifestyle modification education based on health belief model in overweight/obese patients with non-alcoholic fatty liver disease: A parallel randomized controlled clinical trial. Clin. Nutr. ESPEN.

[46] Su R.C., Lad A., Breidenbach J.D., Kleinhenz A.L., Modyanov N., Malhotra D., Haller S.T., Kennedy D.J. (2020). Assessment of diagnostic biomarkers of liver injury in the setting of microcystin-LR (MC-LR) hepatotoxicity. Chemosphere.

[47] Keating S.E., Hackett D.A., George J., Johnson N.A. (2012). Exercise and non-alcoholic fatty liver disease: A systematic review and meta-analysis. J. Hepatol..

[48] Wong V.W.-S., Chan R.S.-M., Wong G.L.-H., Cheung B.H.-K., Chu W.C.-W., Yeung D.K.-W., Chim A.M.-L., Lai J.W.-Y., Li L.S., Sea M.M.-M. (2013). Community-based lifestyle modification programme for non-alcoholic fatty liver disease: A randomized controlled trial. J. Hepatol..

[49] Yang J.S., Tongson J., Kim K.H., Park Y. (2020). Piceatannol attenuates fat accumulation and oxidative stress in steatosis-induced HepG2 cells. Curr. Res. Food Sci..

[50] Ahn S.H., Yang E.S., Cho H.R., Lee S.O., Ha K.T., Kim K. (2020). Herbal formulation MIT ameliorates high-fat diet-induced non-alcoholic fatty liver disease. Integr. Med. Res..

[51] Brunt E.M., Wong V.W., Nobili V., Day C.P., Sookoian S., Maher J.J., Bugianesi E., Sirlin C.B., Neuschwander-Tetri B.A., Rinella M.E. (2015). Nonalcoholic fatty liver disease. Nat. Rev. Dis. Primers..

[52] Bedossa P. (2017). Pathology of non-alcoholic fatty liver disease. Liver Int..

[53] Hernandez A., Reyes D., Geng Y., Arab J.P., Cabrera D., Sepulveda R., Solis N., Buist-Homan M., Arrese M., Moshage H. (2020). Extracellular vesicles derived from fat-laden hepatocytes undergoing chemical hypoxia promote a pro-fibrotic phenotype in hepatic stellate cells. Biochim. Biophys Acta Mol. Basis. Dis..

[54] Zhu L., Baker S.S., Shahein A., Choudhury S., Liu W., Bhatia T., Baker R.D., Lee T. (2018). Upregulation of non-canonical Wnt ligands and oxidative glucose metabolism in NASH induced by methionine-choline deficient diet. Trends Cell Mol. Biol..

[55] Lin H., Zhu L., Baker S.S., Baker R.D., Lee T. (2020). Secreted phosphoglucose isomerase is a novel biomarker of nonalcoholic fatty liver in mice and humans. Biochem. Biophys Res. Commun.

[56] World Health Organization (2014). Age-Standardized Death Rates of Liver Cirrhosis. Global Health Observatory. http://www/.who.int/gho/alcohol/harms_consequences/deaths_liver_cirrhosis/en/index.html.

[57] Zhao J., Nishiumi S., Tagawa R., Yano Y., Inoue J., Hoshi N., Yoshida M., Kodama Y. (2020). Adrenic acid induces oxidative stress in hepatocytes. Biochem. Biophys Res. Commun..

[58] Krawitt E.L. (2006). Autoimmune hepatitis. N. Engl. J. Med..

[59] Czaja A.J. (2017). Global Disparities and Their Implications in the Occurrence and Outcome of Autoimmune Hepatitis. Dig. Dis. Sci..

[60] Ngu J.H., Bechly K., Chapman B.A., Burt M.J., Barclay M.L., Gearry R.B., Stedman C.A. (2010). Population-based epidemiology study of autoimmune hepatitis: A disease of older women?. J. Gastroenterol. Hepatol..

[61] Boberg K.M., Aadland E., Jahnsen J., Raknerud N., Stiris M., Bell H. (1998). Incidence and prevalence of primary biliary cirrhosis, primary sclerosing cholangitis, and autoimmune hepatitis in a Norwegian population. Scand. J. Gastroenterol..

[62] Wang L., Llorente C., Hartmann P., Yang A.M., Chen P., Schnabl B. (2015). Methods to determine intestinal permeability and bacterial translocation during liver disease. J. Immunol. Methods.

[63] Mencin A., Kluwe J., Schwabe R.F. (2009). Toll-like receptors as targets in chronic liver diseases. Gut.

[64] Rao M., Gershon M.D. (2016). The bowel and beyond: The enteric nervous system in neurological disorders. Nat. Rev. Gastroenterol. Hepatol..

[65] Meijers B., Farré R., Dejongh S., Vicario M., Evenepoel P. (2018). Intestinal barrier function in chronic kidney disease. Toxins.

[66] Ramezani A., Massy Z.A., Meijers B., Evenepoel P., Vanholder R., Raj D.S. (2016). Role of the Gut Microbiome in Uremia: A Potential Therapeutic Target. Am. J. Kidney Dis..

[67] Dhiman R.K. (2013). Gut microbiota and hepatic encephalopathy. Metab. Brain. Dis..

[68] Patel V.C., White H., Stoy S., Bajaj J.S., Shawcross D.L. (2016). Clinical science workshop: Targeting the gut-liver-brain axis. Metab. Brain. Dis..

[69] Tranah T.H., Vijay G.K., Ryan J.M., Shawcross D.L. (2013). Systemic inflammation and ammonia in hepatic encephalopathy. Metab. Brain. Dis..

[70] Betrapally N.S., Gillevet P.M., Bajaj J.S. (2016). Changes in the Intestinal Microbiome and Alcoholic and Nonalcoholic Liver Diseases: Causes or Effects?. Gastroenterology.

[71] Bull-Otterson L., Feng W., Kirpich I., Wang Y., Qin X., Liu Y., Gobejishvili L., Joshi-Barve S., Ayvaz T., Petrosino J. (2013). Metagenomic analyses of alcohol induced pathogenic alterations in the intestinal microbiome and the effect of Lactobacillus rhamnosus GG treatment. PLoS ONE.

[72] Chu H., Duan Y., Yang L., Schnabl B. (2019). Small metabolites, possible big changes: A microbiota-centered view of non-alcoholic fatty liver disease. Gut.

[73] Llopis M., Cassard A.M., Wrzosek L., Boschat L., Bruneau A., Ferrere G., Puchois V., Martin J.C., Lepage P., Le Roy T. (2016). Intestinal microbiota contributes to individual susceptibility to alcoholic liver disease. Gut.

[74] Benten D., Wiest R. (2012). Gut microbiome and intestinal barrier failure-The "Achilles heel" in hepatology?. J. Hepatol..

[75] De Minicis S., Rychlicki C., Agostinelli L., Saccomanno S., Candelaresi C., Trozzi L., Mingarelli E., Facinelli B., Magi G., Palmieri C. (2014). Dysbiosis contributes to fibrogenesis in the course of chronic liver injury in mice. Hepatology.

[76] Chen P., Stärkel P., Turner J.R., Ho S.B., Schnabl B. (2015). Dysbiosis-induced intestinal inflammation activates tumor necrosis factor receptor I and mediates alcoholic liver disease in mice. Hepatology.

[77] Feng Y., Teitelbaum D.H. (2013). Tumour necrosis factor--induced loss of intestinal barrier function requires TNFR1 and TNFR2 signalling in a mouse model of total parenteral nutrition. J. Physiol..

[78] Seo B., Jeon K., Moon S., Lee K., Kim W.K., Jeong H., Cha K.H., Lim M.Y., Kang W., Kweon M.N. (2020). Roseburia spp. Abundance Associates with Alcohol Consumption in Humans and Its Administration Ameliorates Alcoholic Fatty Liver in Mice. Cell Host Microb..

[79] Pierantonelli I., Rychlicki C., Agostinelli L., Giordano D.M., Gaggini M., Fraumene C., Saponaro C., Manghina V., Sartini L., Mingarelli E. (2017). Lack of NLRP3-inflammasome leads to gut-liver axis derangement, gut dysbiosis and a worsened phenotype in a mouse model of NAFLD. Sci. Rep..

[80] Teltschik Z., Wiest R., Beisner J., Nuding S., Hofmann C., Schoelmerich J., Bevins C.L., Stange E.F., Wehkamp J. (2012). Intestinal bacterial translocation in rats with cirrhosis is related to compromised Paneth cell antimicrobial host defense. Hepatology.

[81] Kang D.J., Betrapally N.S., Ghosh S.A., Sartor R.B., Hylemon P.B., Gillevet P.M., Sanyal A.J., Heuman D.M., Carl D., Zhou H. (2016). Gut microbiota drive the development of neuroinflammatory response in cirrhosis in mice. Hepatology.

[82] Petrasek J., Iracheta-Vellve A., Csak T., Satishchandran A., Kodys K., Kurt-Jones E.A., Fitzgerald K.A., Szabo G. (2013). STING-IRF3 pathway links endoplasmic reticulum stress with hepatocyte apoptosis in early alcoholic liver disease. Proc. Natl. Acad. Sci. USA.

[83] Koop D.R., Klopfenstein B., Iimuro Y., Thurman R.G. (1997). Gadolinium chloride blocks alcohol-dependent liver toxicity in rats treated chronically with intragastric alcohol despite the induction of CYP2E1. Mol. Pharmacol..

[84] Douhara A., Moriya K., Yoshiji H., Noguchi R., Namisaki T., Kitade M., Kaji K., Aihara Y., Nishimura N., Takeda K. (2015). Reduction of endotoxin attenuates liver fibrosis through suppression of hepatic stellate cell activation and remission of intestinal permeability in a rat non-alcoholic steatohepatitis model. Mol. Med. Rep..

[85] Tedesco D., Thapa M., Chin C.Y., Ge Y., Gong M., Li J., Gumber S., Speck P., Elrod E.J., Burd E.M. (2018). Alterations in Intestinal Microbiota Lead to Production of Interleukin 17 by Intrahepatic gammadelta T-Cell Receptor-Positive Cells and Pathogenesis of Cholestatic Liver Disease. Gastroenterology.

[86] Soderborg T.K., Clark S.E., Mulligan C.E., Janssen R.C., Babcock L., Ir D., Young B., Krebs N., Lemas D.J., Johnson L.K. (2018). The gut microbiota in infants of obese mothers increases inflammation and susceptibility to NAFLD. Nat. Commun..

[87] Rainer F., Horvath A., Sandahl T., Leber B., Schmerboeck B., Blesl A., Groselj-Strele A., Stauber R., Fickert P., Stiegler P. (2018). Soluble CD 163 and soluble mannose receptor predict survival and decompensation in patients with liver cirrhosis, and correlate with gut permeability and bacterial translocation. Aliment. Pharmacol. Ther..

[88] Bajaj J.S., Kakiyama G., Zhao D., Takei H., Fagan A., Hylemon P., Zhou H., Pandak W.M., Nittono H., Fiehn O. (2017). Continued alcohol misuse in human cirrhosis is associated with an impaired gut–liver axis. Alcohol. Clin. Exp. Res..

[89] Schäfer C., Parlesak A., Schütt C., Christian Bode J., Bode C. (2002). Concentrations of lipopolysaccharide-binding protein, bactericidal/permeability-increasing protein, soluble CD14 and plasma lipids in relation to endotoxaemia in patients with alcoholic liver disease. Alcohol Alcohol..

[90] Yuan J., Baker S.S., Liu W., Alkhouri R., Baker R.D., Xie J., Ji G., Zhu L. (2014). Endotoxemia unrequired in the pathogenesis of pediatric nonalcoholic steatohepatitis. J. Gastroenterol. Hepatol..

[91] Luther J., Garber J.J., Khalili H., Dave M., Bale S.S., Jindal R., Motola D.L., Luther S., Bohr S., Jeoung S.W. (2015). Hepatic injury in nonalcoholic steatohepatitis contributes to altered intestinal permeability. Cell. Mol. Gastroenterol. Hepatol..

[92] Miele L., Marrone G., Lauritano C., Cefalo C., Gasbarrini A., Day C., Grieco A. (2013). Gut-liver axis and microbiota in NAFLD: Insight pathophysiology for novel therapeutic target. Curr. Pharm. Des..

[93] Zhu L., Baker S.S., Gill C., Liu W., Alkhouri R., Baker R.D., Gill S.R. (2013). Characterization of gut microbiomes in nonalcoholic steatohepatitis (NASH) patients: A connection between endogenous alcohol and NASH. Hepatology.

[94] Riva A., Patel V., Kurioka A., Jeffery H.C., Wright G., Tarff S., Shawcross D., Ryan J.M., Evans A., Azarian S. (2018). Mucosa-associated invariant T cells link intestinal immunity with antibacterial immune defects in alcoholic liver disease. Gut.

[95] Lin R., Zhou L., Zhang J., Wang B. (2015). Abnormal intestinal permeability and microbiota in patients with autoimmune hepatitis. Int. J. Clin. Exp. Pathol..

[96] Maccioni L., Gao B., Leclercq S., Pirlot B., Horsmans Y., De Timary P., Leclercq I., Fouts D., Schnabl B., Starkel P. (2020). Intestinal permeability, microbial translocation, changes in duodenal and fecal microbiota, and their associations with alcoholic liver disease progression in humans. Gut Microb..

[97] Zeng W., Shen J., Bo T., Peng L., Xu H., Nasser M.I., Zhuang Q., Zhao M. (2019). Cutting edge: Probiotics and fecal microbiota transplantation in immunomodulation. J. Immunol. Res..

[98] Craven L., Rahman A., Nair Parvathy S., Beaton M., Silverman J., Qumosani K., Hramiak I., Hegele R., Joy T., Meddings J. (2020). Allogenic Fecal Microbiota Transplantation in Patients With Nonalcoholic Fatty Liver Disease Improves Abnormal Small Intestinal Permeability: A Randomized Control Trial. Am. J. Gastroenterol..

[99] Raj A.S., Shanahan E.R., Tran C.D., Bhat P., Fletcher L.M., Vesey D.A., Morrison M., Holtmann G., Macdonald G.A. (2019). Dysbiosis of the Duodenal Mucosal Microbiota Is Associated With Increased Small Intestinal Permeability in Chronic Liver Disease. Clin. Transl. Gastroenterol..

[100] Wei Y., Li Y., Yan L., Sun C., Miao Q., Wang Q., Xiao X., Lian M., Li B., Chen Y. (2020). Alterations of gut microbiome in autoimmune hepatitis. Gut.

[101] Goeser F., Munch P., Lesker T.R., Lutz P.L., Kramer B., Kaczmarek D.J., Finnemann C., Nischalke H.D., Geffers R., Parcina M. (2020). Neither black nor white: Do altered intestinal microbiota reflect chronic liver disease severity?. Gut.

[102] Chen Y., Ji F., Guo J., Shi D., Fang D., Li L. (2016). Dysbiosis of small intestinal microbiota in liver cirrhosis and its association with etiology. Sci. Rep..

[103] Xu M., Wang B., Fu Y., Chen Y., Yang F., Lu H., Chen Y., Xu J., Li L. (2012). Changes of fecal Bifidobacterium species in adult patients with hepatitis B virus-induced chronic liver disease. Microb. Ecol..

[104] Heidrich B., Vital M., Plumeier I., Doscher N., Kahl S., Kirschner J., Ziegert S., Solbach P., Lenzen H., Potthoff A. (2018). Intestinal microbiota in patients with chronic hepatitis C with and without cirrhosis compared with healthy controls. Liver Int..

[105] Nier A., Engstler A.J., Maier I.B., Bergheim I. (2017). Markers of intestinal permeability are already altered in early stages of non-alcoholic fatty liver disease: Studies in children. PLoS ONE.

[106] Schwimmer J.B., Johnson J.S., Angeles J.E., Behling C., Belt P.H., Borecki I., Bross C., Durelle J., Goyal N.P., Hamilton G. (2019). Microbiome Signatures Associated with Steatohepatitis and Moderate to Severe Fibrosis in Children With Nonalcoholic Fatty Liver Disease. Gastroenterology.

[107] Giorgio V., Miele L., Principessa L., Ferretti F., Villa M.P., Negro V., Grieco A., Alisi A., Nobili V. (2014). Intestinal permeability is increased in children with non-alcoholic fatty liver disease, and correlates with liver disease severity. Dig. Liver Dis..

[108] Colakoglu M., Xue J., Trajkovski M. (2020). Bacteriophage Prevents Alcoholic Liver Disease. Cell.

[109] Duan Y., Llorente C., Lang S., Brandl K., Chu H., Jiang L., White R.C., Clarke T.H., Nguyen K., Torralba M. (2019). Bacteriophage targeting of gut bacterium attenuates alcoholic liver disease. Nature.

[110] Leclercq S., Matamoros S., Cani P.D., Neyrinck A.M., Jamar F., Starkel P., Windey K., Tremaroli V., Backhed F., Verbeke K. (2014). Intestinal permeability, gut-bacterial dysbiosis, and behavioral markers of alcohol-dependence severity. Proc. Natl. Acad. Sci. USA.

[111] Lang S., Duan Y., Liu J., Torralba M.G., Kuelbs C., Ventura-Cots M., Abraldes J.G., Bosques-Padilla F., Verna E.C., Brown R.S. (2020). Intestinal Fungal Dysbiosis and Systemic Immune Response to Fungi in Patients With Alcoholic Hepatitis. Hepatology.

[112] Younossi Z.M., Ratziu V., Loomba R., Rinella M., Anstee Q.M., Goodman Z., Bedossa P., Geier A., Beckebaum S., Newsome P.N. (2019). Obeticholic acid for the treatment of non-alcoholic steatohepatitis: Interim analysis from a multicentre, randomised, placebo-controlled phase 3 trial. Lancet.

[113] Staels B., Rubenstrunk A., Noel B., Rigou G., Delataille P., Millatt L.J., Baron M., Lucas A., Tailleux A., Hum D.W. (2013). Hepatoprotective effects of the dual peroxisome proliferator-activated receptor alpha/delta agonist, GFT505, in rodent models of nonalcoholic fatty liver disease/nonalcoholic steatohepatitis. Hepatology.

[114] Musso G., Cassader M., Gambino R. (2016). Non-alcoholic steatohepatitis: Emerging molecular targets and therapeutic strategies. Nat. Rev. Drug Discov..

[115] Ratziu V., Ladron-De-Guevara L., Safadi R., Poordad F., Fuster F., Flores-Figueroa J., Harrison S.A., Arrese M., Fargion S., Ben-Bashat D. (2018). One-year results of the global phase 2b randomized placebo-controlled arrest trial of aramchol, a stearoyl CoA desaturase inhibitor, in patients with NASH. Proc. Hepatol..

[116] Joo J.S., Cho S.Y., Rou W.S., Kim J.S., Kang S.H., Lee E.S., Moon H.S., Kim S.H., Sung J.K., Kwon I.S. (2020). TEAD2 as a novel prognostic factor for hepatocellular carcinoma. Oncol. Rep..

[117] Kirk A.P., Jain S., Pocock S., Thomas H.C., Sherlock S. (1980). Late results of the Royal Free Hospital prospective controlled trial of prednisolone therapy in hepatitis B surface antigen negative chronic active hepatitis. Gut.

[118] Soloway R.D., Summerskill W., Baggenstoss A.H., Geall M.G., Gitnick G.L., Elveback L.R., Schoenfield L.J. (1972). Clinical, biochemical, and histological remission of severe chronic active liver disease: A controlled study of treatments and early prognosis. Gastroenterology.

[119] Summerskill W.H., Korman M.G., Ammon H.V., Baggenstoss A.H. (1975). Prednisone for chronic active liver disease: Dose titration, standard dose, and combination with azathioprine compared. Gut.

[120] Hammad A., Kaido T., Uemoto S. (2015). Perioperative nutritional therapy in liver transplantation. Surg. Today.

[121] Safari Z., Monnoye M., Abuja P.M., Mariadassou M., Kashofer K., Gerard P., Zatloukal K. (2019). Steatosis and gut microbiota dysbiosis induced by high-fat diet are reversed by 1-week chow diet administration. Nutr. Res..

[122] Soares J.B., Pimentel-Nunes P., Roncon-Albuquerque R., Leite-Moreira A. (2010). The role of lipopolysaccharide/toll-like receptor 4 signaling in chronic liver diseases. Hepatol. Int..

[123] Yang W.S., Zeng X.F., Liu Z.N., Zhao Q.H., Tan Y.T., Gao J., Li H.L., Xiang Y.B. (2020). Diet and liver cancer risk: A narrative review of epidemiological evidence. Br. J. Nutr..

[124] Sheng L., Jena P.K., Hu Y., Liu H.X., Nagar N., Kalanetra K.M., French S.W., French S.W., Mills D.A., Wan Y.Y. (2017). Hepatic inflammation caused by dysregulated bile acid synthesis is reversible by butyrate supplementation. J. Pathol..

[125] Singh V., Yeoh B.S., Chassaing B., Xiao X., Saha P., Aguilera Olvera R., Lapek J.D., Zhang L., Wang W.B., Hao S. (2018). Dysregulated Microbial Fermentation of Soluble Fiber Induces Cholestatic Liver Cancer. Cell.

[126] Keshavarzian A., Choudhary S., Holmes E.W., Yong S., Banan A., Jakate S., Fields J.Z. (2001). Preventing gut leakiness by oats supplementation ameliorates alcohol-induced liver damage in rats. J. Pharmacol. Exp. Ther..

[127] Cho Y.E., Kim D.K., Seo W., Gao B., Yoo S.H., Song B.J. (2019). Fructose Promotes Leaky Gut, Endotoxemia, and Liver Fibrosis Through Ethanol-Inducible Cytochrome P450-2E1-Mediated Oxidative and Nitrative Stress. Hepatology.

[128] Munukka E., Pekkala S., Wiklund P., Rasool O., Borra R., Kong L., Ojanen X., Cheng S.M., Roos C., Tuomela S. (2014). Gut-adipose tissue axis in hepatic fat accumulation in humans. J. Hepatol..

[129] Ferolla S.M., Couto C.A., Costa-Silva L., Armiliato G.N., Pereira C.A., Martins F.S., Ferrari Mde L., Vilela E.G., Torres H.O., Cunha A.S. (2016). Beneficial Effect of Synbiotic Supplementation on Hepatic Steatosis and Anthropometric Parameters, But Not on Gut Permeability in a Population with Nonalcoholic Steatohepatitis. Nutrients.

[130] Horvath A., Leber B., Schmerboeck B., Tawdrous M., Zettel G., Hartl A., Madl T., Stryeck S., Fuchs D., Lemesch S. (2016). Randomised clinical trial: The effects of a multispecies probiotic vs. placebo on innate immune function, bacterial translocation and gut permeability in patients with cirrhosis. Aliment. Pharmacol. Ther..

[131] Nier A., Brandt A., Rajcic D., Bruns T., Bergheim I. (2019). Short-Term Isocaloric Intake of a Fructose- but not Glucose-Rich Diet Affects Bacterial Endotoxin Concentrations and Markers of Metabolic Health in Normal Weight Healthy Subjects. Mol. Nutr. Food Res..

[132] Biolato M., Manca F., Marrone G., Cefalo C., Racco S., Miggiano G.A., Valenza V., Gasbarrini A., Miele L., Grieco A. (2019). Intestinal permeability after Mediterranean diet and low-fat diet in non-alcoholic fatty liver disease. World J. Gastroenterol.

[133] Damms-Machado A., Louis S., Schnitzer A., Volynets V., Rings A., Basrai M., Bischoff S.C. (2017). Gut permeability is related to body weight, fatty liver disease, and insulin resistance in obese individuals undergoing weight reduction. Am. J. Clin. Nutr..

[134] Chen M., Hui S., Lang H., Zhou M., Zhang Y., Kang C., Zeng X., Zhang Q., Yi L., Mi M. (2019). SIRT3 Deficiency Promotes High-Fat Diet-Induced Nonalcoholic Fatty Liver Disease in Correlation with Impaired Intestinal Permeability through Gut Microbial Dysbiosis. Mol. Nutr. Food Res..

[135] Hu E.D., Chen D.Z., Wu J.L., Lu F.B., Chen L., Zheng M.H., Li H., Huang Y., Li J., Jin X.Y. (2018). High fiber dietary and sodium butyrate attenuate experimental autoimmune hepatitis through regulation of immune regulatory cells and intestinal barrier. Cell Immunol..

[136] Vieira A.T., Fukumori C., Ferreira C.M. (2016). New insights into therapeutic strategies for gut microbiota modulation in inflammatory diseases. Clin. Transl. Immunol..

[137] Mendes M.C.S., Paulino D.S., Brambilla S.R., Camargo J.A., Persinoti G.F., Carvalheira J.B.C. (2018). Microbiota modification by probiotic supplementation reduces colitis associated colon cancer in mice. World J. Gastroenterol..

[138] Forsyth C.B., Farhadi A., Jakate S.M., Tang Y., Shaikh M., Keshavarzian A. (2009). Lactobacillus GG treatment ameliorates alcohol-induced intestinal oxidative stress, gut leakiness, and liver injury in a rat model of alcoholic steatohepatitis. Alcohol.

[139] Cui Y., Qi S., Zhang W., Mao J., Tang R., Wang C., Liu J., Luo X.M., Wang H. (2019). Lactobacillus reuteri ZJ617 culture supernatant attenuates acute liver injury induced in mice by lipopolysaccharide. J. Nutr..

[140] Ritze Y., Bardos G., Claus A., Ehrmann V., Bergheim I., Schwiertz A., Bischoff S.C. (2014). Lactobacillus rhamnosus GG protects against non-alcoholic fatty liver disease in mice. PLoS ONE.

[141] Kwak D.S., Jun D.W., Seo J.G., Chung W.S., Park S.E., Lee K.N., Khalid-Saeed W., Lee H.L., Lee O.Y., Yoon B.C. (2014). Short-term probiotic therapy alleviates small intestinal bacterial overgrowth, but does not improve intestinal permeability in chronic liver disease. Eur. J. Gastroenterol. Hepatol..

[142] Mencarelli A., Cipriani S., Renga B., Bruno A., D’Amore C., Distrutti E., Fiorucci S. (2012). VSL#3 resets insulin signaling and protects against NASH and atherosclerosis in a model of genetic dyslipidemia and intestinal inflammation. PLoS ONE.

[143] Li M., Zhu L., Xie A., Yuan J. (2015). Oral administration of Saccharomyces boulardii ameliorates carbon tetrachloride-induced liver fibrosis in rats via reducing intestinal permeability and modulating gut microbial composition. Inflammation.

[144] Scorletti E., Afolabi P.R., Miles E.A., Smith D.E., Almehmadi A., Alshathry A., Childs C.E., Del Fabbro S., Bilson J., Moyses H.E. (2020). Synbiotics Alter Fecal Microbiomes, But Not Liver Fat or Fibrosis, in a Randomized Trial of Patients With Nonalcoholic Fatty Liver Disease. Gastroenterology.

[145] Pedersen B.K., Saltin B. (2015). Exercise as medicine-evidence for prescribing exercise as therapy in 26 different chronic diseases. Scand. J. Med. Sci. Sports.

[146] Rodriguez B., Torres D.M., Harrison S.A. (2012). Physical activity: An essential component of lifestyle modification in NAFLD. Nat. Rev. Gastroenterol. Hepatol..

[147] European Association for the Study of the L., European Association for the Study of D., European Association for the Study of O. (2016). EASL-EASD-EASO Clinical Practice Guidelines for the Management of Non-Alcoholic Fatty Liver Disease. Obes. Facts.

[148] Thyfault J.P., Rector R.S. (2020). Exercise Combats Hepatic Steatosis: Potential Mechanisms and Clinical Implications. Diabetes.

[149] Liu Y., Wang Y., Ni Y., Cheung C.K.Y., Lam K.S.L., Wang Y., Xia Z., Ye D., Guo J., Tse M.A. (2020). Gut Microbiome Fermentation Determines the Efficacy of Exercise for Diabetes Prevention. Cell Metab..

[150] Kantartzis K., Thamer C., Peter A., Machann J., Schick F., Schraml C., Konigsrainer A., Konigsrainer I., Krober S., Niess A. (2009). High cardiorespiratory fitness is an independent predictor of the reduction in liver fat during a lifestyle intervention in non-alcoholic fatty liver disease. Gut.

[151] Mailing L.J., Allen J.M., Buford T.W., Fields C.J., Woods J.A. (2019). Exercise and the Gut Microbiome: A Review of the Evidence, Potential Mechanisms, and Implications for Human Health. Exerc. Sport Sci. Rev..

[152] Keirns B.H., Koemel N.A., Sciarrillo C.M., Anderson K.L., Emerson S.R. (2020). Exercise and Intestinal Permeability: Another Form of Exercise-Induced Hormesis?. Am. J. Physiol. Gastrointest Liver Physiol..

[153] Hawley J.A. (2020). Microbiota and muscle highway-two way traffic. Nat. Rev. Endocrinol..

[154] Clark A., Mach N. (2016). Exercise-induced stress behavior, gut-microbiota-brain axis and diet: A systematic review for athletes. J. Int. Soc. Sports Nutr..

[155] Mach N., Fuster-Botella D. (2017). Endurance exercise and gut microbiota: A review. J. Sport Health Sci..

[156] Oseini A.M., Sanyal A.J. (2017). Therapies in non-alcoholic steatohepatitis (NASH). Liver Int..

[157] Lee J., Hong S.W., Rhee E.J., Lee W.Y. (2012). GLP-1 Receptor Agonist and Non-Alcoholic Fatty Liver Disease. Diabetes. Metab. J..

[158] Dietrich P., Hellerbrand C. (2014). Non-alcoholic fatty liver disease, obesity and the metabolic syndrome. Best Pract. Res. Clin. Gastroenterol..

[159] Review T., LaBrecque D.R., Abbas Z., Anania F., Ferenci P., Khan A.G., Goh K.L., Hamid S.S., Isakov V., Lizarzabal M. (2014). World Gastroenterology Organisation global guidelines: Nonalcoholic fatty liver disease and nonalcoholic steatohepatitis. J. Clin. Gastroenterol..

[160] Neuschwander-Tetri B.A., Loomba R., Sanyal A.J., Lavine J.E., Van Natta M.L., Abdelmalek M.F., Chalasani N., Dasarathy S., Diehl A.M., Hameed B. (2015). Farnesoid X nuclear receptor ligand obeticholic acid for non-cirrhotic, non-alcoholic steatohepatitis (FLINT): A multicentre, randomised, placebo-controlled trial. Lancet.

[161] Adorini L., Pruzanski M., Shapiro D. (2012). Farnesoid X receptor targeting to treat nonalcoholic steatohepatitis. Drug Discov. Today.

[162] Arya A.K., Hu B. (2018). Brain-gut axis after stroke. Brain. Circ..

[163] Kohler C.A., Maes M., Slyepchenko A., Berk M., Solmi M., Lanctot K.L., Carvalho A.F. (2016). The Gut-Brain Axis, Including the Microbiome, Leaky Gut and Bacterial Translocation: Mechanisms and Pathophysiological Role in Alzheimer’s Disease. Curr. Pharm. Des..

[164] Srikantha P., Mohajeri M.H. (2019). The Possible Role of the Microbiota-Gut-Brain-Axis in Autism Spectrum Disorder. Int. J. Mol. Sci..

[165] Dutta S.K., Verma S., Jain V., Surapaneni B.K., Vinayek R., Phillips L., Nair P.P. (2019). Parkinson’s Disease: The Emerging Role of Gut Dysbiosis, Antibiotics, Probiotics, and Fecal Microbiota Transplantation. J. Neurogastroenterol. Motil..

[166] Slyepchenko A., Maes M., Jacka F.N., Kohler C.A., Barichello T., McIntyre R.S., Berk M., Grande I., Foster J.A., Vieta E. (2017). Gut Microbiota, Bacterial Translocation, and Interactions with Diet: Pathophysiological Links between Major Depressive Disorder and Non-Communicable Medical Comorbidities. Psychother. Psychosom..

[167] Kelly J.R., Kennedy P.J., Cryan J.F., Dinan T.G., Clarke G., Hyland N.P. (2015). Breaking down the barriers: The gut microbiome, intestinal permeability and stress-related psychiatric disorders. Front. Cell Neurosci..

[168] Karakula-Juchnowicz H., Dzikowski M., Pelczarska A., Dzikowska I., Juchnowicz D. (2016). The brain-gut axis dysfunctions and hypersensitivity to food antigens in the etiopathogenesis of schizophrenia. Psychiatr. Pol..

[169] Herpertz-Dahlmann B., Seitz J., Baines J. (2017). Food matters: How the microbiome and gut-brain interaction might impact the development and course of anorexia nervosa. Eur. Child. Adolesc. Psychiatry.

[170] Leung D.H., Yimlamai D. (2017). The intestinal microbiome and paediatric liver disease. Lancet Gastroenterol. Hepatol..

